# Microtubules in cell migration

**DOI:** 10.1042/EBC20190016

**Published:** 2019-07-29

**Authors:** Clare Garcin, Anne Straube

**Affiliations:** 1 Centre for Mechanochemical Cell Biology, University of Warwick, Coventry CV4 7AL, U.K.; 2 Division of Biomedical Sciences, Warwick Medical School, University of Warwick, Coventry CV4 7AL, U.K.

**Keywords:** cell adhesion, cell migration, integrins, kinesins, microfilaments, microtubule

## Abstract

Directed cell migration is critical for embryogenesis and organ development, wound healing and the immune response. Microtubules are dynamic polymers that control directional migration through a number of coordinated processes: microtubules are the tracks for long-distance intracellular transport, crucial for delivery of new membrane components and signalling molecules to the leading edge of a migrating cell and the recycling of adhesion receptors. Microtubules act as force generators and compressive elements to support sustained cell protrusions. The assembly and disassembly of microtubules is coupled to Rho GTPase signalling, thereby controlling actin polymerisation, myosin-driven contractility and the turnover of cellular adhesions locally. Cross-talk of actin and microtubule dynamics is mediated through a number of common binding proteins and regulators. Furthermore, cortical microtubule capture sites are physically linked to focal adhesions, facilitating the delivery of secretory vesicles and efficient cross-talk. Here we summarise the diverse functions of microtubules during cell migration, aiming to show how they contribute to the spatially and temporally coordinated sequence of events that permit efficient, directional and persistent migration.

## The properties of microtubules

**Figure 1:**
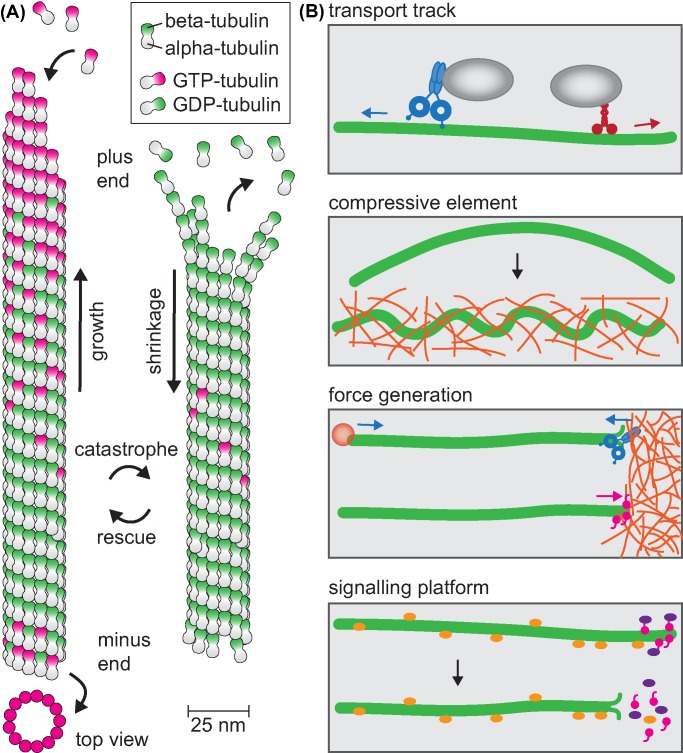
Microtubule structure and functions (**A**) Microtubules are 25-nm diameter tubes assembled from 13 protofilaments of head-to-tail arranged heterodimers of α-tubulin and β-tubulin. Microtubules assemble primarily at their ends by addition of GTP-bound tubulin dimers that gradually hydrolyse GTP once incorporated in the lattice. The presence of a GTP-tubulin stabilises growth phases, loss of the cap results in catastrophe and the microtubule shrinks until it is rescued. (**B**) Overview of microtubule functions: transport tracks for minus end-directed dynein and predominantly plus end-directed kinesins, the stiffness of microtubules paired with viscosity of the cytoplasm allows microtubules to resist large compressive forces, microtubule assembly and disassembly results in pushing and pulling forces can be coupled to perform work, microtubules serve as signalling hubs by sequestering lattice-bound signalling molecules or enriching signalling complexes in the plus end complex, these are released upon depolymerisation.

Microtubules, together with actin and intermediate filaments, are the major components of the cytoskeleton. Microtubules are hollow filaments with a diameter of 25 nm constructed from α-tubulin and β-tubulin heterodimers (Figure 1A). Because microtubules are much wider than actin (6 nm) and intermediate filaments (10–12 nm), they are also much stiffer. Microtubules have a persistence length of several millimetres [1–3], which is approximately 200-times larger than that of actin (15–20 µm) [3,4]. While it takes approximately 1 pN force to buckle a single microtubule, within the cytoplasm microtubules are surrounded by the meshwork of other cytoskeletal components that limit sideways motion and thus allow microtubules to resist compressive loads in the order of 100 pN [5]. This enables microtubules to provide structural support for the cell and form relatively straight, long tracks for long-distance intracellular transport (Figure 1B). Microtubules have intrinsic polarity as they are built from tubulin dimers in a head-to-tail fashion. This results in two different ends, with the plus end (where β-tubulin is exposed) assembling faster than the minus end (where α-tubulin is exposed) (Figure 1A). Moreover, the uniform dimer orientation in the microtubule lattice allows molecular motors to move directionally along microtubules and thus mediate efficient, processive long-distance transport of cellular cargoes. Finally, their unique dynamic properties enable microtubules to explore the entire cellular space as they undergo continuous phases of polymerisation (growth) and depolymerisation (shrinkage) and transitions between these states predominantly occur near the cell boundaries [6,7]. Microtubules are often observed to undergo repeated catastrophe (switching from growth to shrinkage) and rescue (switching from shrinkage to growth) events near the cell cortex [8]. This enables the repeated targeting of focal adhesions and other cortical sites, but might also serve signalling functions that depend on the localised formation and release of the microtubule +TIP (microtubule plus-end tracking protein) complex. The +TIP complex is a dynamic network of proteins that assembles at the ends of growing microtubules, regulating microtubule dynamics and interactions of microtubules with the cell cortex and other subcellular structures (reviewed in [9]). The core of the +TIP network is formed by the end-binding proteins EB1, EB2 and EB3, that recognise the nucleotide state of tubulin at the growing end and mediate binding of a large number of proteins containing CAP-Gly domains, SxIP or LxxPTPh motives [10–14]. Thus, microtubule ends are platforms for protein interactions that are coupled to their dynamic state.

## Overview of microtubule functions in cell migration

Cell migration is a plastic process that employs different modes depending on the cellular environment. The main parameters are confinement and adhesiveness. In a physically confined space with low adhesion, cells predominantly move in an amoeboid manner by extruding membrane blebs. This is largely driven by myosin-mediated contractility [15,16]. In contrast, mesenchymal migration requires cell protrusions generated by actin polymerisation and traction forces transmitted to the extracellular matrix or neighbouring cells using cell–substrate or cell–cell adhesions [17,18]. Especially in larger cell types, microtubules play important roles in mesenchymal migration [19].

These roles include providing an intracellular transport network for the rapid and directed transport of membrane vesicles, signalling molecules, RNAs and other cytoskeletal components, which are essential to maintain polarity and directionally persistent cell migration. Microtubules also contribute to the formation and maintenance of membrane protrusions through their ability to resist high compressive loads and generate pushing forces [20,21]. The additional ability of microtubules to exert pulling forces is employed by migrating cells to move the nucleus forwards and position the centrosome [22]. The asymmetric organisation of the microtubule network is key to the front/back polarity of cells as it allows differential regulation of intracellular events at the leading edge versus the cell rear. Finally, microtubules play a role in signalling during cell migration. Microtubules sequester and inactivate signalling molecules that are released and activated upon microtubule depolymerisation. The microtubule +TIP complex assembling at growing microtubule ends concentrates signalling molecules and actin assembly factors. Thereby microtubule dynamics is coupled to the regulation of actin dynamics, Rho GTPase signalling and the regulation of focal adhesion turnover. The diverse roles played by microtubules during cell migration are summarised in Figure 1B.

## Microtubule-based transport

The targeted delivery of membranes, mRNAs and polarity factors to the leading edge supports cell protrusion. Microtubules enable this transport by providing tracks for molecular motors of the kinesin superfamily. The human genome encodes 45 kinesins, approximately half of which are plus-end directed transporters and are thus capable of mediating the efficient translocation of secretory vesicles and mRNAs from the cell centre to the cell boundaries (reviewed in [23]). As the plasma membrane can only stretch by a few percent [24], a supply of newly synthesised lipids and/or localised exocytosis of secretory vesicles is required to generate extra cell surface area for cell protrusions [25,26]. In migrating fibroblasts, polarised exocytosis of secretory vesicles towards the leading edge of the cell depends on the microtubule network and kinesin-1 [27–29]. The contents of these vesicles can include secretory proteins, integrins for cellular adhesion or new membrane components [30,31].

A number of specific mRNAs undergo microtubule-based directional transport towards the leading edge. There are several advantages of localised translation, including facilitated protein complex formation, and reduced energy expenditure compared with re-localising larger proteins [32,33]. Of particular interest are mRNAs encoding actin regulators, such as the components of the actin nucleating Arp2/3 complex, which rely on microtubules for their localisation [34]. Profilin mRNA is also transported along microtubules towards sites of rapid actin polymerisation, such as the leading edge [35]. Profilin ‘charges’ actin monomers with ATP, enabling them to participate in filament assembly [36,37]. mRNA encoding β-actin also localises to the leading edge in numerous cell types and appears to rely on microtubules to be transported there [38–42]. Localised translation of actin at the leading edge contributes to the large demand of globular actin for rapid polymerisation that is estimated to incorporate approximately 3.6 × 10^6^ actin molecules per minute in metastatic MTLn3 cells [43]. Therefore, the localised translation of actin and actin regulators likely contributes to directional migration by limiting the region where efficient actin polymerisation can occur to the leading edge.

**Figure 2:**
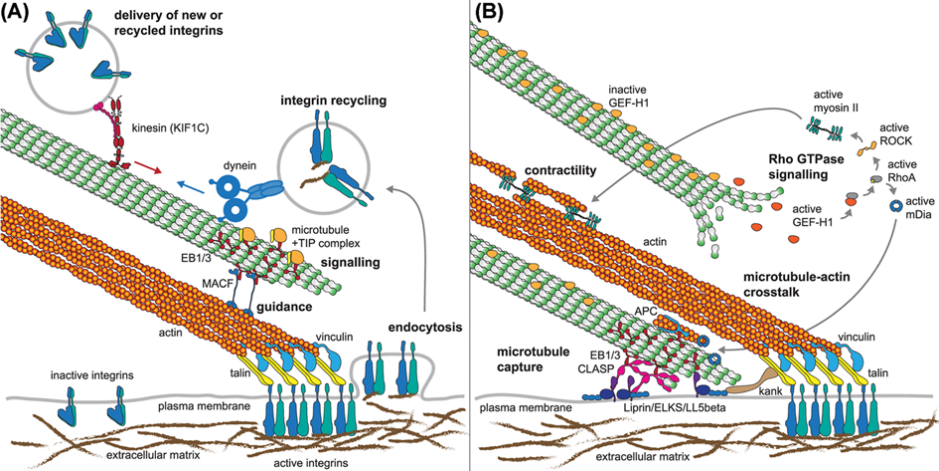
Examples of microtubule–actin cross-talk and regulation of focal adhesions (**A**) Focal adhesions link the extracellular matrix via transmembrane receptors (integrins) via talin (yellow) to actin filaments (orange). Vinculin (blue) binds talin and actin and reinforces tension within focal adhesions. Recycling and new delivery of integrins and other adhesion receptors depend on directional transport on microtubules using KIF1C and dynein. Guidance of microtubule assembly along actin fibres mediated by EB1/EB3 (red) that recognise growing microtubule ends and MACF (blue) that link EBs to actin. EBs also mediate the formation of signalling complexes at microtubule plus ends and deliver relaxation factors to focal adhesions. (**B**) Microtubules are captured at cortical sites near focal adhesions through a complex involving CLASPs (magenta), LL5β (purple), ELKS and Liprin (blue). These are linked to focal adhesions via Kank proteins (brown). CLASPs stimulate microtubule rescues to keep microtubule ends close to the cortical capture site. APC and mDia (blue) cooperate to nucleate actin filaments and also stabilise microtubules. Microtubules regulate Rho GTPases signalling locally, for example by sequestering GEF-H1, which is activated upon its release. GEF-H1 in turn activates RhoA, which stimulates contractility through myosin II and actin assembly through mDia. Abbreviations: APC, adenomatous polyposis coli; GEF-H1, guanine nucleotide exchange factor-H1.

Microtubule-based transport is also important for the recycling of integrins within migrating cells (Figure 2). Integrins are the major components of focal adhesion complexes, which link the cytoskeleton to the extracellular substrate and allow the transmission of traction forces [44]. Focal adhesions are mechanosensitive clutches, that are reinforced if the contact site is under tension [45]. Microtubules are a key regulator triggering the turnover of focal adhesions [46–48], but are equally important for their force-dependent maturation by ensuring a ready supply of integrin molecules [49]. The kinesin Kif1C transports integrin-containing vesicles along microtubules, which is particularly relevant for the maintenance of trailing adhesions in cell tails and permitting directionally persistent migration [50]. KIF1C also transports the protein tyrosine phosphatase PTPN21/PTPD1, which regulates focal adhesions through activation of Src kinase and focal adhesion kinase FAK [51,52]. Further, kinesin KIF15 promotes the internalisation of integrins by recruiting Dab2, a clathrin-associated sorting protein, to the plasma membrane, thereby promoting integrin endocytosis and recycling [53]. In contrast, kinesin-1 has been implicated in the microtubule-mediated dissolution of mature focal adhesions [47,54], potentially by transporting metalloprotease MT1-MMP or other factors that act as relaxation factors to release cortical forces at focal adhesions and allow their rapid disassembly [55–58].

Microtubule-based transport of mitochondria controls the energy supply within migrating cells. Cells migrating in an amoeboid manner, such as leucocytes, localise their mitochondria within the uropod, a microtubule-filled protrusion at the cell rear that is important particularly for directional cell migration [59,60]. Fibroblasts and cancer cells, on the other hand, employ microtubule-based transport to localise their mitochondria towards the leading edge of the cell to meet the higher energy demand in cell protrusions [61].

It is clear that the microtubule network provides a means by which specific cellular components can be transported rapidly and directionally to where they are required during cell migration. The bias in microtubule transport is achieved in two ways: an asymmetric organisation of microtubules largely due to front-directed microtubules emanating from the Golgi [62] and the PTM (post-translational modification) of tubulin resulting in subsets of microtubules that are preferred tracks for particular molecular motors. For example, kinesin-1 preferentially moves along polyglutamylated and acetylated microtubules while kinesin-3 KIF1C is negatively regulated by tubulin acetylation and kinesin-2 requires detyrosination of α-tubulin [63–65].

## Microtubules as force generators in cell migration

Microtubules themselves are motors in the sense that both the polymerisation and depolymerisation of microtubules can perform work. Microtubules assemble from GTP-bound tubulin dimers. GTP is gradually hydrolysed to GDP once the dimer is buried in the microtubule lattice and some of the energy is stored in the lattice as strain [66,67]. It is generally thought that the bulk of the pushing forces required for cell protrusion are generated by actin polymerisation, however, evidence for a contribution of microtubules to cell protrusion is growing. For example, in astrocytes, a dense array of microtubules reaches right to the leading edge and is essential for cell protrusion [68]. Neuronal cells employ a kinesin-1-powered microtubule sliding mechanism to generate the pushing forces required for neurite outgrowth [69]. This process is facilitated by the depolymerisation of actin, which is in line with the finding that actin severing by cofilin allows microtubules to populate a protrusion [70]. Invasion of mesenchymal cells in 3D culture requires persistent microtubule assembly to push out and support long protrusions [71]. Microtubules deform the membrane at the tip of protrusions and undergo extensive buckling, suggesting that microtubules are under high compressive forces. The persistent microtubule assembly required for protrusion requires the cooperation of microtubule stabilising factors SLAIN2-chTOG and CLASP1 (cytoplasmic linker associated protein 1) [71].

## Cross-talk of actin and microtubule dynamics

As mentioned above, microtubules contribute to the assembly and regulation of the actin cytoskeleton by delivering mRNAs encoding actin and actin-regulators. However, the interplay of these two filament systems is more intimate. The list of microtubule dynamics regulators that are found to also bind to and regulate actin assembly is continuously growing and indeed growing microtubule ends can serve as platforms for actin filament nucleation. APC (adenomatous polyposis coli) stabilises microtubules and directly nucleates actin filaments both on its own and in synergy with the formin mDia1 [72–76]. APC binds to the microtubule +TIP complex via EB1 and accumulates at the leading edge of migrating cells with the help of kinesin-1 and kinesin-2 [77,78]. Therefore, the microtubule-dependent deposition of APC may regulate the spatial organisation of actin within migrating cells. mDia1 is also recruited to growing microtubule ends via CLIP-170 (cytoplasmic linker protein 170) [79]. Together, CLIP-170 and mDia form an actin barbed-end tracking complex that can dramatically accelerate actin elongation. In the presence of growing microtubules and EB1, the CLIP-170/mDia1 complex mediates the rapid assembly of actin filaments from the microtubule tip [80]. As CLIP-170, mDia and APC also stabilise microtubules [72,76,81], the cross-talk of microtubule and actin dynamics is evident. Furthermore, microtubule–actin cross-linking factor MACF1/ACF7 has an N-terminal actin-binding calponin-homology domain, a C-terminal SxIP motif and a Gas2-related microtubule-binding domain, and can therefore physically link both cytoskeletal systems and bind to EB proteins [13,82,83]. Microtubule dynamics and organisation is perturbed, when ACF7/MACF1 no longer guides microtubule assembly along actin bundles and fails to capture microtubule plus ends at actin-rich cortical sites [84]. CLASPs, microtubule rescue factors that localise to the distal ends of microtubules, and MAP4 (microtubule-associated protein 4), a microtubule lattice binder and stabiliser, have also been shown to bind actin [85,86]. CLASP2 localises to actin stress fibres, and *in vitro* experiments suggest that MAP4 is capable of promoting actin bundling. Profilin localises to microtubule plus ends and regulates both actin and microtubule dynamics at the leading edge [87]. Microtubule dynamics at the leading edge may also be regulated by EB1, the core component of microtubule plus tip complex, which has both a microtubule-binding domain and an actin-binding domain that partially overlap. This means that EB1 can bind to both cytoskeletal components but not simultaneously [88]. Thus it is possible that EB1 is sequestered in regions of high actin concentration (such as at the leading edge), which might destabilise the microtubule +TIP complex and induce microtubule catastrophe. In line with this, photo-dissociation experiments inducing the loss of EB1 and EB3-dependent cargoes from microtubule plus ends attenuates microtubule growth and cell protrusion [89].

In turn, microtubule organisation is influenced by actin. The retrograde flow of actin in lamellipodia results in a continuous backward transport of microtubules, often resulting in their buckling and breaking [90–93]. Microtubule access to the lamellipodium is facilitated through the action of cofilin [70]. Interestingly, cofilin can also bind to microtubules directly [94], suggesting that microtubules might be able to direct the actin depolymerising and severing activity of cofilin and thereby create space for themselves in dense actin networks. Actin is also known to guide microtubule growth towards the leading edge [95]. The bifunctional spektraplakins MACF1/ACF7 and Dystonin as well as Gas2-like proteins mediate dynamic interactions of the growing microtubule end via EB1 and EB3 with actin filaments [84,96–99] (Figure 2A). This guidance mechanism ensures efficient targeting of microtubules to cellular adhesion sites, where actin filaments terminate. Microtubule targeting of focal adhesions also involves the CLASP-mediated capture of microtubule ends at cortical sites via LL5β, ELKS and liprin, which in turn link via adapters Kank1 and Kank2 to talin, a key mechanical element in focal adhesions linking integrins to actin filaments [100–102] (Figure 2B).

## Microtubule-dependent regulation of cellular adhesions

Cells adhere and crawl either directly on other cells or the extracellular matrix. Cell–cell contacts are largely mediated by cadherins [103], while cell–substrate adhesions are usually mediated by integrins [104]. Both types of adhesions are linked to actin filaments to transduce mechanical forces [105,106] and are targeted and regulated by microtubules [107,108]. Adhesions at the cell front are required to transmit protrusion forces from actin polymerisation to the environment and to anchor contractile actomyosin fibres to haul the cell body forward [109]. Adhesions at the cell rear are important to establish and maintain cell polarity. Both cadherin-mediated cell–cell contacts and integrin-mediated cell–substrate contracts at the cell rear facilitate directional migration as cell protrusion opposes the rear drag forces [50,110]. Similarly to ensure the efficient recycling and supply of integrins at the cell surface [50], microtubules also transport N-cadherin and p120 catenin to cell–cell junctions [111,112].

Microtubules target focal adhesions repeatedly and trigger their dissolution [48,54,108,113,114]. As discussed above, this involves the kinesin-dependent delivery of a proposed relaxation factor [54,108]. One possible cargo is APC, which is transported to the cell edge by kinesin-1 and kinesin-2 [78,115,116], where it may promote focal adhesion dynamics [117]. Indeed, a mutant of APC that can still bind microtubules, but no longer nucleate actin results in severe defects in microtubule-mediated focal adhesion turnover [74]. Components of the microtubule +TIP complex that assemble at growing microtubule ends, have also been implicated in mediating focal adhesion turnover. MAP4K4 (mitogen-activated protein kinase 4) is thought to be delivered to focal adhesions via the +TIP protein EB2 and regulate focal adhesion dissolution by activation of Arf6, which in turn promotes integrin internalisation [118] (Figure 2A). A third mechanism is the microtubule-mediated suppression of contractility in the vicinity of focal adhesions through sequestering of GEF-H1 (guanine nucleotide exchange factor (GEF)-H1), which results in their relaxation. If microtubule capture at focal adhesions is disturbed, GEF-H1 release activates myosin, which results in turn stimulates force-dependent maturation of focal adhesions [101].

## Microtubules and Rho GTPase signalling

GEF-H1 is a Rho GEF that binds to the microtubule lattice and is inactive in its microtubule-bound form [119,120]. Microtubule depolymerisation leads to the release and activation of GEF-H1. This will convert RhoA into its GTP-bound, active form and the subsequent activation of Rho-associated kinase ROCK that phosphorylates and thereby activates myosin light chain [120,121] (Figure 2B). Therefore microtubule depolymerisation activates non-muscle myosin II, increases contractility and the stabilisation of focal adhesions. Other Rho GEFs also bind to microtubules [122], ensuring redundancy and robustness in the microtubule depolymerisation-induced activation of Rho signalling. Activation of RhoA stimulates the formation of contractile actomyosin fibres and reinforcement of focal adhesions. In contrast, microtubule polymerisation activates Rac1 signalling and thereby actin-mediated cell protrusion via Rac GEFs STEF/Tiam2 and TRIO [123–125].

In turn, Rac1 promotes pioneer behaviour in microtubules and stimulates their assembly at the leading edge of the cell through the activation of CLASPs [126,127]. RhoA signalling also stabilises microtubules via activation of mDia and the inhibition of stathmin [128–130]. Stathmin is a potent microtubule destabiliser that acts by sequestering tubulin in an assembly-incompetent configuration and forms an activity gradient in motile cells [131].

## Asymmetric organisation of microtubules

For microtubules to regulate polarised cell protrusion, cell adhesion and contractility to result in efficient and directionally persistent cell migration, microtubules need to be organised asymmetrically in migrating cells.

In many migrating cells, the centrosome precedes the nucleus and this used to be considered a major factor for cell polarity and the formation of a front-biased microtubule network (reviewed in [132,133]). One reason proposed for this is the role of centrosome-anchored microtubules to position the Golgi in the cell centre, which in turn organises a subset of Golgi-derived microtubules that are polarised to the leading edge of the cell [134]. However, the centrosome position relative to the nucleus is dependent on the geometry of the cell and leucocytes or fibroblasts migrating in 3D tend to position the centrosome behind the nucleus [135–138]. Indeed, recent work indicates that the centrosome nucleates a radial array of microtubules, which is unsuitable to support the polarity of a migrating cell and highlights the importance of non-centrosomal microtubules, the majority of which are usually associated with the Golgi [139].

Beyond asymmetric nucleation/anchorage of microtubules, differences in microtubule stability contribute to a polarised microtubule cytoskeleton in motile cells. For example, the differential localisation of kinesins within migrating cells has been implicated in the differential regulation of microtubule dynamics. While the kinesin-4 Kif4 stabilises microtubules at the cell front [140], the kinesin-13 Kif2C/MCAK triggers microtubule disassembly preferentially at the cell rear [141]. A number of microtubule +TIPs have been implicated in ‘capturing’ microtubules at the leading edge. Clusters of APC at the tips of cellular protrusions stabilise microtubule tips [76] and CLASPs keep microtubule tips associated with cortical microtubule attachment sites near focal adhesions via the interaction with LL5β [100,102]. Captured microtubules remain dynamic, but undergo frequent switches between growth and shrinkage [142]. However, the longevity of the microtubule lattice behind the tip is thought to result in the accumulation of PTMs of tubulin. We have mentioned above that PTMs affect molecular motors and thus mark subsets of microtubules as preferred tracks for intracellular transport of certain motor–cargo combinations. PTMs can affect the properties of microtubules and their ability to resist mechanical load directly – the acetylation of α-tubulin at Lys^40^ in the lumen of the microtubule, confers higher resistance to mechanical damage of the microtubule lattice [143]. Furthermore, PTMs regulate the affinity to microtubule-associated proteins, which in turn can exacerbate the differences in microtubule stability. For example, adding a side chain of glutamic acids to the C-terminal tails of tubulin regulates the susceptibility of microtubules for spastin, a microtubule severing enzyme [144], while removing the C-terminal tyrosine from α-tubulin reduces the association of the kinesin-13s KIF2A and KIF2C/MCAK, both potent microtubule depolymerisers [145]. This so-called tubulin code is just being deciphered [146] and will undoubtedly play an important role in the maintenance of a polarised microtubule network and its asymmetric regulation of the cell migration machinery.

## Concluding remarks

While once considered a purely actin-based process, the importance of microtubules for directing and regulating cell migration is increasingly appreciated. In the next decade, major advances are expected in understanding the mechanisms of the actin–microtubule cross-talk that involves many shared regulators and dynamic linkers as well as the mechanisms that allow the maintenance of microtubule polarity to support directionally persistent cell migration and at the same time a rapid rearrangement when environmental signals favour cell turning.

## Summary

- Microtubules and their associated motors deliver mRNA, secretory vesicles, integrins and signalling molecules to the leading edge of migrating cells.
- Microtubules can withstand high compressive loads and generate pushing and pulling forces to support cell protrusion and positioning of the nucleus and other organelles.
- Microtubules and actin dynamics are coordinated by a number of shared binders and regulators. Actin guides growing microtubules towards cellular adhesion complexes. Repeated targeting of focal adhesions by microtubules triggers their disassembly.
- Microtubule assembly stimulates Rac1 signalling, while depolymerisation stimulates RhoA signalling via microtubule-associated guanine exchange factors.
- Microtubules are organised asymmetrically in migrating cells to enable them to fulfil distinct functions at the front and rear of the cell.

## Abbreviations

APC: adenomatous polyposis coli
CLASP: cytoplasmic linker associated protein
CLIP-170: cytoplasmic linker protein 170
EB: end-binding protein
FAK: focal adhesion kinase
GEF: guanine nucleotide exchange factor
GEF-H1: guanine nucleotide exchange factor-H1
MACF1: microtubule-actin crosslinking factor 1
MAP4: microtubule-associated protein 4
MCAK: mitotic centromer-associated kinesin
MT1-MMP: membrane-associated matrix metalloproteinase
PTM: post-translational modification
ROCK: Rho-associated coiled-coil-containing protein kinase
